# Examining nurses' understanding and knowledge about preparation for COVID-19 in Ardabil hospitals in Iran

**DOI:** 10.1186/s12913-024-10826-2

**Published:** 2024-03-07

**Authors:** Milad Minehmorad, Reza Nemati-Vakilabad, Mohammad Badpeyma, Alireza Mirzaei

**Affiliations:** 1 Students Research Committee, School of Nursing and Midwifery, Ardabil University of Medical Sciences, Ardabil, Iran; 2 Student Research Committee, School of Nursing and Midwifery, Guilan University of Medical Sciences, Rasht, Iran; 3 Department of Emergency Nursing, School of Nursing and Midwifery, Ardabil University of Medical Sciences, Ardabil, Iran

**Keywords:** Outbreak, Preparedness, Pandemic, Nurse, Iran

## Abstract

**Aims:**

The purpose of this study was to evaluate the preparedness of Iranian nurses for potential pandemics.

**Background:**

Nurses play a critical role in managing pandemics. They require adequate training, proper equipment, and organizational support to be well-prepared.

**Methods:**

A descriptive cross-sectional study was conducted in Ardabil, Iran, from July to September 2023, involving 233 nurses from five hospitals. The number of nurses required for each hospital was calculated based on the proportion of nurses in each hospital. Data was collected through a paper-based form that included information about the participants' demographic characteristics and their level of pandemic preparedness in health services. The collected data was analyzed using descriptive statistics to determine the demographic characteristics and levels of pandemic preparedness. Pearson's test was also conducted to establish a relationship between different dimensions of pandemic preparedness.

**Results:**

Most participants relied on clinical measures and supported using human resources and environmental methods to curb the transmission of a pandemic. They felt assured in their ability to explain the preventive measures against the pandemic. However, fewer respondents had access to healthcare improvement programs, and only a few worked remotely from home.

**Conclusions:**

According to our study, 90.1% of nurses believe hand washing is the most effective way to prevent spreading infections. Additionally, healthcare professionals can use various tools to respond to the pandemic, including screening for COVID-19 at work, health and wellness programs, telecommuting, COVID-19 Safe programs, social media, and posters. Nurses need continuous education in hand hygiene, health programs, remote work options, and pandemic-safe programs to control infections, reduce risks, and optimize patient care during the pandemic.

## Introduction

In late 2019, a new strain of coronavirus, SARS-CoV-2, emerged and rapidly spread globally. On March 11, 2020, it was declared a pandemic [1]. The COVID-19 pandemic has impacted more than 185 countries worldwide, affecting 80% of the global population. Iran has also experienced multiple outbreaks [2]. The World Health Organization (WHO) declared the new coronavirus outbreak the sixth global public health emergency, threatening all countries worldwide [2]. Although the disease has resulted in deaths, its impact on people's health has gone beyond that [3].

The pandemic has overwhelmed hospitals, reduced capacity, exhausted treatment teams, and increased the risk of infection among staff. The healthcare sector needs more resources, careful planning, and a review of the COVID-19 management approach [3–5]. According to scientific evidence, a significant part of cases of contracting the disease of COVID-19 related to occupational exposures have been reported [6]. The Centers for Disease Control and Prevention (CDC) guidelines acknowledge that healthcare workers were on the front lines of the fight against COVID-19 and were also exposed to more significant contamination [7]. Properly managing this global epidemic and providing safe and effective services required that hospitals be adequately prepared to work their clinical services [7]. A study in Iran revealed that hospitals accepting COVID-19 patients encountered several challenges [8]. These challenges included accurately diagnosing patients with diseases, having sufficient resources to care for more patients in a surge in COVID-19 cases, continuously monitoring and evaluating the quality-of-care services provided, and establishing effective communication channels within and outside the hospital [9, 10]. The lack of adequate and correct knowledge of how the disease spreads leads to the lack of preparation for its control and the contamination of medical personnel [11]. It is essential to determine how the virus that causes the disease spreads, how long it takes to incubate, and the duration of the infection in those who are sick. This information can help predict the necessary measures for controlling disease in the workplace [11, 12]. In preparing for a pandemic like COVID-19, it is vital to create a plan addressing employees' and hospitals' strengths and weaknesses [13].

Preparedness of hospitals against crises is the primary condition for dealing with an epidemic of disease because if hospitals are vulnerable, society will suffer a more significant crisis, and the treatment of patients in out-of-hospital environments will bring human disaster [11]. In pandemics, including the COVID-19 disease, hospitals' resistance to the disease is more critical because hospital employees themselves are dangerous agents of disease transmission and crisis and are also at risk, affecting the severity and scope of the pandemic [12].

The pandemic, the newly emerging COVID-19 disease, has shown that despite the development and access to medical equipment and financial resources, the governments could have performed better in dealing with a new unknown disease. Until effective medicine to control and treat the disease becomes available, the infrastructure must be physical facilities, including hospitals and health centers, and human resources should prepare themselves against this crisis [13]. Therefore, the preparation of hospitals in dealing with crises from various aspects, including management, communication, human resources, supply of drugs and equipment, and other diagnostic, treatment, and support services needed by patients, companions, and healthcare workers from a variety of indicators It is considered the development of health and treatment of the country [11].

Planning and preparing adequately to minimize unnecessary expenses and resource wastage in the healthcare sector during a pandemic is essential. Identifying factors that affect healthcare worker preparedness and providing them with better support and education can enhance their response to crises, ultimately reducing the impact on public health and safety. This study aimed to assess the preparedness of Iranian nurses for possible pandemic outbreaks.

## Methods

### Purpose of the study

This study aimed to assess the preparedness of Iranian nurses for possible pandemic outbreaks.

### Study design

A cross-sectional descriptive study was conducted in Ardabil province following STROBE guidelines.

### Participants

This study focuses on nurses working at five hospitals in Ardabil, Iran: Imam Khomeini, Fatemi, Alavi, Bu Ali, and Imam Reza. The study's statistical population comprises people who possess a bachelor's degree or higher and have at least six months of work experience in the clinical departments of these hospitals. Nurses who work in non-clinical or administrative departments and those who refused to participate were excluded from the study. The samples were selected from the five hospitals using convenience sampling because of easy accessibility and staff cooperation. As per Kline's formula [14], the sample size for this study should be 2.5 to 5 times the number of instrument materials. The study requires at least 200 samples that include 87 items, out of which 15 items are related to the demographic questionnaire, and 72 are associated with the pandemic preparedness questionnaire. Therefore, at least 218 people should participate in the research (by multiplying 87 by 2.5). To account for possible withdrawals of 10% and lost samples, a sample size of 239 individuals was determined. However, six questionnaires were excluded from the study due to incomplete data recording. The number of nurses required for each hospital was calculated based on the proportion of nurses in each hospital. The study included 528 participants from Imam Hospital, 184 from Fatemi Hospital, 126 from Alavi Hospital, 96 from Bu Ali Hospital, and 86 from Imam Reza Hospital. Between July and September 2023, stratified random sampling was used to select 123 participants from Imam Hospital, 42 from Fatemi Hospital, 30 from Alavi Hospital, 23 from Bu Ali Hospital, and 21 from Imam Reza Hospital.

### Data collection

The researchers handed out paper questionnaires to eligible nurses at Ardabil City Hospital during different shifts. The nurses were informed that their participation in the study was voluntary and they could withdraw at any point if they wished to do so. Two questionnaires were used to collect information in this study.

### Demographic characteristics form

The demographic characteristics form included questions about age, gender, education, place of residence, marital status, employment, income, and workplace.

### Pandemic preparedness in health services scale

This tool was initially developed by McGill et al. in Australia to evaluate the readiness of healthcare workers against pandemics such as COVID-19 [15]. The questionnaire comprises 32 questions, including internationally validated items from epidemic survey tools. It was prepared based on the study population and is divided into five sub-groups that cover clinical, communication, environment, human resources, and general preparation. The questionnaire uses a six-point Likert scale, ranging from strongly disagree to strongly agree, with an option for undecided/not sure or "not applicable". The items in the questionnaire assess the respondents' opinions on general preparedness and their knowledge of health service preparedness strategies, particularly in terms of their effectiveness in helping prepare or protect against a COVID-19 outbreak at work. The questionnaire groups similar aspects of preparation into five scales, and its reliability was calculated in McGill's study [15] using Cronbach's alpha score of 0.71. After obtaining written permission from the tool manufacturer via email, the readiness questionnaire was translated into Persian independently and separately by two people fluent in Persian and English. Then, 12 Ardabil University of Medical Sciences faculty members were asked to evaluate the questionnaire's content validity index (CVI) and content validity ratio (CVR). Using a four-part spectrum, the experts assessed each question separately based on simplicity, appropriateness, and certainty. The content validity index was 0.92, indicating a high content validity level. In addition, the questionnaire showed good reliability with Cronbach's alpha coefficient of 0.91. To analyze the characteristics.

### Data analysis

The data was analyzed using the Statistical Package for the Social Sciences (SPSS) version 22.0 software. Descriptive statistics were used to analyze the demographic characteristics and pandemic preparedness levels. Pearson's test was conducted to determine the correlation between different aspects of pandemic preparedness. This statistical analysis helped identify the primary factors contributing to pandemic preparedness and develop appropriate interventions to enhance overall preparedness levels. The normal distribution of the dataset was assessed using tests in SPSS, such as the Kolmogorov–Smirnov and Shapiro–Wilk.

## Results

The data from 233 nurses was analyzed. Most participants were female (187, 80.3%) and married (163, 70.0%). In addition, most of them were formal (133, 57.1%) with a bachelor's degree (216, 92.7%). Their average work experience was 8.53 years, with a standard deviation of 6.125. The average age of the participants was 32.90 years, with a standard deviation of 6.75 years. Most nurses worked in general wards (68, 29.2%) and had received personal protective equipment (PPE) training (128, 54.9%). For more information, please refer to Table 1 for participant profiles.

**Table 1:** Demographic characteristic all participant (*n* = 233)

Vriable	Type	Mean	SD
Age		32.90	6.75
Work Experience		8.53	6.12
		*n*	%
Gender	Male	46	19.7
	Female	187	80.3
Department	General	68	29.2
	ICU	42	18.0
	Surgical	34	14.6
	Children	17	7.3
	Emergency	55	23.6
	Infection	17	7.3
Hospital	Emam Khomeini	114	48.9
	Fatemi	35	15.0
	Bou Ali	36	15.5
	Alavi	26	11.2
	Emam Reza	22	9.4
Marital	Single	70	30.0
	Married	163	70.0
Employment	Projective	62	26.6
	Partial contractual	18	7.7
	Contractual	20	8.6
	Official	133	57.1
Education	Bachelor	216	92.7
	Master	17	7.3
PPE Education	*Yes*	128	54.9
	*No*	105	45.1
Response	*Yes*	130	55.8
	*No*	103	44.2
Personal Response	*Yes*	160	68.7
	*No*	73	31.3
Less work	*Yes*	30	12.9
	*No*	203	87.1
Isolation department	*Yes*	29	12.4
	*Not sure*	69	29.6
	*No*	135	57.9

According to the results of Table 2, it was found that most of the respondents used various clinical measures such as hand washing (90.1%), using alcohol disinfectants (88%), using surgical masks (85.8%) and N95 (75.1%), gloves. (81.1%), clothes (67.8%), protective glasses (70.8%), and antiviral drugs (55.4%) agreed or very much agreed. Also, most respondents stated that there is an infection control committee in their hospital (86.7%). However, fewer participants have said that the hospital has the necessary preparation to deal with the spread of coronavirus (51.1%). Also, fewer of them had received the flu vaccine and believed that antiviral drugs were necessary.

**Table 2:** Clinical measures for pandemic survey scale items and responses (*n* = 233)

Scale	Question	Item	Agreement (agree/ strongly agree responses) Scale Question Item Number (n)	(%)
Clinical measures	Do you believe that the following clinical measures are useful in protecting you from contracting/being? infected with coronavirus (COVID-19) at work?	a. Hand washing	210	90.1
		b. Alcohol rubs/hand sanitizer	205	88.0
		c. Surgical mask (face mask)	200	85.8
		d. N95 mask (respirator mask)	175	75.1
		e. Gloves	189	81.1
		f. Gowns	158	67.8
		g. Goggles	165	70.8
		h. Antiviral drugs	129	55.4
	Please indicate your level of agreement with the following statements	a. There is an infection control committee at my hospital	202	86.7
		b. I have been recommended by the hospital to receive a flu vaccination (‘flu shot’)	174	74.7
		c. There are infection control staff in my hospital	205	88.0
		d. My hospital has a plan for a coronavirus (COVID-19) outbreak	119	51.1
		e. My hospital has developed/adopted clinical guidelines for responding to a coronavirus (COVID-19) outbreak	131	56.2
	Please indicate your level of agreement with the following statements. In the past 3 months:	a. I have attended infection control training sessions	156	67.0
		b. I have participated in infection control audits	145	62.2
		c. I have attended infection control related meetings	142	60.9
		d. I have received a flu vaccination (‘flu shot’)	121	51.9
		e. I have someone to turn to if I have a problem in using personal protective equipment (PPE)	146	62.7

According to the results of Table 3, most respondents agreed or strongly agreed with using various human resources methods such as body temperature checks, screening tests for COVID-19 patients without symptoms, working from home, and flexible leave planning. Also, fewer respondents stated they were familiar with, had access to, and used the [Health Service] Health and Care Improvement Program for [Health Service] employees. However, few nurses confirmed that they were aware of the health service's COVID-19 health and wellness program for staff. They also stated that they needed more access to this program. Also, only some of them worked at home.

**Table 3:** Human resources for pandemic survey scale items and responses (*n* = 233)

Scale	Question	Item	Agreement (agree/ strongly agree responses) Scale Question Item Number (n)	(%)
Human resources	Do you believe that the following human resources related strategies are useful in supporting? you/protecting you from coronavirus (COVID-19) at work?	a. Temperature checks (for employees)	134	57.5
		b. Asymptomatic COVID-19 screening tests for employees	176	75.5
		c. Employees working from home	109	46.8
		d. Flexible leave planning	165	70.8
	Please indicate your level of agreement with the following statements	I am aware of [HEALTH SERVICE]’s COVID-19 Health and Wellbeing program for employees	84	36.1
		b. I have accessed the [HEALTH SERVICE]’s COVID-19 Health and Wellbeing program for employees	80	34.3
		c. I found it helpful to access the [HEALTH SERVICE]’s COVID-19 Health and Wellbeing program for employees	142	60.9

In Table 4, most respondents used various communication and environmental methods such as posters and information notices, CEO video updates, information related to COVID-19 in the internal network [health service], and social network updates. [Health service] and discussed. Meetings agree or strongly agree on COVID-19. Also, most respondents use different environmental methods such as limiting visitors, checking body temperature, social distancing, limiting standard work tools, separating areas, increasing exhaust air flow, hygiene and disinfection of workshops/offices, and reducing the number of work sessions. Are site and telephone meetings agreed upon or strongly agreed upon? But fewer decided to email COVID-19 notifications and updates.

**Table 4:** Communications and environment for pandemic survey scale items and responses

Scale	Question	Item	Agreement (agree/ strongly agree responses) Scale Question Item Number (n)	(%)
**Communications**	Do you believe that the following communication strategies are useful in preparing you to respond? to coronavirus (COVID-19) at work?	a. Information posters/notices [3]	165	70.8
		b. COVID-19 email updates	99	42.1
		c. Video updates from the CEO regarding coronavirus (COVID-19)	151	64.8
		d. Information on the [HEALTH SERVICE] intranet regarding coronavirus (COVID-19)	164	70.4
		e. [HEALTH SERVICE] social media updates regarding coronavirus (COVID-19)	179	76.8
		f. Updates from the [HEALTH SERVICE] Department Heads regarding coronavirus (COVID-19)	162	69.5
		g. Meetings discussing coronavirus (COVID-19)	142	60.9
		h. Downloading the COVID Safe app	179	76.8
**Environment**	Do you believe that the following environmental strategies are useful in protecting you from? contracting/being infected with coronavirus (COVID-19) at work?	a. Limiting visitors to the [HEALTH SERVICE] [3]	202	86.7
		b. Temperature checks (for patients and visitors) [3]	162	69.5
		c. social distancing at work	200	85.8
		d. Limiting shared desks/workstations (“hot-desks”)	205	88.0
		e. Area isolation (Restricting employee/visitor access to areas) [3]	205	88.0
		f. Increasing airflow through work areas	197	84.5
		g. Additional cleaning/sanitization of work areas/offices	160	68.7
		h. Reduced frequency of work meetings	137	58.8
		i. Telehealth sessions (limiting face-to-face work with patients)	153	65.6
		j. Limiting clinical placements at the [Health Service]	176	75.5

According to the results of Table 5, most of the participants were confident in explaining the pandemic outbreak methods to colleagues, patients, and society. They believe the health service's decisions were based on the best available evidence and the appropriate response. Participants learned as much as possible to deal with the risk of contracting the virus at work. However, some fear contracting the virus in case of an outbreak and may consider taking time off. Participants think coworkers with cold/flu symptoms should stay home. Some feel that the precautions could be more worthwhile. However, fewer respondents felt that their hospital was prepared for a pandemic and that they were involved in decision-making. Some are concerned about the risk of infection at work and are considering other career options. There are also concerns about potential surges in the work.

**Table 5:** General preparedness for pandemic survey scale items and responses (*n* = 233)

Scale	Question	Item	Agreement (agree/ strongly agree responses) Scale Question Item Number (n)	(%)
General preparedness	Please indicate your level of agreement with the following statements	a. My hospital is well prepared to respond to a coronavirus (COVID-19) outbreak	85	36.5
		b. I have been involved in decision-making regarding coronavirus (COVID-19) preparation in my role/area at the [Health Service]	61	25.8
		c. I am well prepared to respond to a coronavirus (COVID-19) outbreak in my role at the [Health Service]	84	36.1
		d. I am confident in explaining coronavirus (COVID-19) procedures to colleagues at work	121	51.9
		e. I am confident in explaining coronavirus (COVID-19) pandemic to patients	199	85.4
		f. I am confident in explaining coronavirus (COVID-19) pandemic to members of the community	202	86.7
	Please indicate your level of agreement with the following statements	a. I believe decisions regarding coronavirus (COVID-19) preparation at the [HEALTH SERVICE] were based on the best available evidence at the time	188	80.7
		b. I believe the scale of the [Health Service]’s response to coronavirus (COVID-19) preparation was appropriate for the level of potential risk/threat	182	78.1
		c. I have personally coped with the threat of a coronavirus (COVID-19) outbreak at work by learning as much as I can about it	148	63.5
		d. I would accept the risk of contracting coronavirus (COVID-19) at work in the event of an outbreak	155	66.5
		e. I am afraid of falling ill with coronavirus (COVID-19) in the event of an outbreak at work	119	51.1
		f. I am worried about a second wave of coronavirus (COVID-19) outbreak at work	88	37.8
		g. I might consider taking extended leave from my role because of the risk of contracting coronavirus (COVID-19) at work	162	69.5
		h. I might look for another job or consider resigning because of the risk of contracting coronavirus (COVID-19) at work [	66	28.3
		i. My colleagues should stay home from work if they have cold/flu symptoms during the coronavirus (COVID-19) pandemic	142	49.9
		j. I feel it is pointless to take precautions regarding coronavirus (COVID-19) at work	158	67.8

Table 6 shows the correlation between the different scores of the Pandemic Preparedness Survey. This survey has determined that higher scores in clinical criteria, human resources, communication, environment, and overall preparedness correlate positively and significantly. The correlation between human resources and clinical measures is higher than other variables. In short, an increase in the score of one variable corresponds to an increase in the score of other variables.

**Table 6:** Correlation between scores of Preparedness for pandemic subscales (*n* = 233)

Variable	1	2	3	4	5
Clinical measures	1	0.540^a^	0.281^a^	0.495^a^	0.407^a^
Human resources	0.540^a^	1	0.356^a^	0.511^a^	0.499^a^
Communications	0.281^a^	0.356^a^	1	0.425^a^	0.273^a^
Environment	0.495^a^	0.511^a^	0.425^a^	1	0.337^a^
General preparedness	0.407^a^	0.499^a^	0.273^a^	0.337^a^	1

1 Clinical measures, 2 Human resources, 3 Communications, 4 Environment, 5 General preparedness

^a^Correlation is significant at the 0.01 level (2-tailed)

## Discussion

Nurses play a crucial role in handling outbreaks and ensuring patient safety. This study in Iran assessed their preparedness to manage epidemics by examining their knowledge, skills, and equipment. This study aimed to evaluate Iranian nurses' preparedness level for potential pandemics.

Based on the findings of this study, nurses ranked the highest in hand washing as a clinical measure to prevent the spread of infections, scoring 90.1%. They also believe that hand washing is more effective than other methods in preventing the transmission of viral infections. These findings are consistent with previous studies conducted among nurses in Australia and Taiwan [15, 16]. However, they do not align with a study conducted among nurses and paramedics in Libya [17]. The study found fewer nurses would receive the flu vaccine during the pandemic. This is consistent with similar studies conducted among nurses in Hong Kong and healthcare workers in Poland [18–20]. The main reason behind this reluctance was the lack of accurate information about the vaccine and its effectiveness at the beginning of the pandemic. However, with more information about the safety and efficacy of vaccines and more deaths from COVID-19, many nurses have changed their attitude and are now actively participating in the vaccination process.

Our study found that screening nurses for COVID-19 was the most effective way to prevent the spread of the virus in their workplace. This finding aligns with a study conducted by Al Baalharith et al. in Saudi Arabia, which also found a similar connection between screening and prevention [21]. The importance of screening tests is highlighted in the studies conducted by Rivett et al., as they can help diagnose individuals with or without symptoms [22]. Carl et al.'s comments suggest that the results of screening tests can provide valuable insights into the spread of the virus, as there is a correlation between positive results of RT-PCR and the prevalence of COVID-19 in the community [23]. These studies emphasize the need to follow comprehensive screening protocols. However, the limitations of current screening methods, especially the lower sensitivity of some screening tests such as CT scan and PCR, require continuous research and the development of more accurate and sensitive diagnostic tools.

It is essential to recognize the importance of health and wellness programs for employees, particularly those in the healthcare industry, especially during the COVID-19 pandemic. A study by Korman et al. has highlighted that implementing health and wellness programs can significantly improve healthcare workers' diagnoses and performance. These finding contrasts earlier assumptions that such programs are unnecessary [24]. Furthermore, a study by Bailey et al. reported that most respondents experienced increased stress and reduced sleep due to COVID-19 [25]. Thus, wellness programs targeting depression and anxiety can help alleviate burnout and manage the psychological impact of COVID-19. Healthcare workers must utilize these programs to maintain their well-being. Unfortunately, nurses' lack of knowledge about these programs highlights a communication gap. More efforts are needed to raise awareness and promote such programs among healthcare professionals.

During the pandemic, telecommuting was utilized as a measure to decrease the spread of the coronavirus. However, a recent study by some nurses suggests that it is ineffective to prevent the pandemic. Hughes et al. showed that remote work enhances productivity and enables nurses to complete administrative tasks without distractions [26]. Working from home can be comfortable and stress-free, but it also carries potential risks that must be considered to prevent the spread of COVID-19 [27]. Working from home is one effective measure to prevent the spread of COVID-19 and protect nurses during pandemics. It is essential to follow personal and social safety guidelines, such as using protective equipment, maintaining social distance, and controlling workplace traffic. It is also crucial to ensure adequate ventilation and disinfection systems are in place to maintain a healthy work environment during a pandemic.

The COVID Safe program was an essential tool in the fight against COVID-19. It allows users to share their call history and receive notifications if they encounter someone who has tested positive for the virus. Nurses also used this app to respond to the pandemic and help infected people properly. Studies have confirmed that the COVID-19 program has effectively spread information and trace the virus [28]. However, its effectiveness depended on user compliance and the availability of testing for epidemic control [29].

Nurses are often active users of social media platforms, particularly in critical areas like COVID-19, where public health is rapidly evolving [30]. A survey by Chan et al. found that medical professionals use social media for education, showing it's an effective way to give nurses and healthcare workers timely information [31]. Social media platforms, posters, and informational notices are effective ways to share concise information about COVID-19 with healthcare professionals, including nurses. Visual aids like posters can effectively convey information about COVID-19, using clear language and graphic representations to increase understanding and retention of information [32]. Also, the study by Tripathi et al. showed that disseminating visual information, including posters, can effectively raise awareness and influence behavior [33]. Posters and information notices are practical tools for sharing COVID-19 information among healthcare professionals. Although email updates are helpful, nurses report receiving less information through email. High volumes of emails can lead to information overload, reducing the likelihood of retaining information. Iranian nurses prefer online resources for acquiring strategies to deal with Corona.

It is essential to limit the use of shared desks or "hot desks" to prevent spreading infectious diseases such as COVID-19. Most nurses believe that reducing the crowding of workstations is a critical measure in preventing the virus from spreading. A study conducted by Dancer et al. demonstrated that hot desking is one of the most effective strategies in reducing the spread of respiratory viruses, including influenza, among hospital employees [34]. The study concluded that reducing the number of people using a workstation can significantly reduce the risk of cross-contamination and the subsequent spread of viruses.

Christie et al. conducted a study on the opinions of nurses regarding gathering at the workstation. They surveyed nurses in Nursing Standard to examine their attitudes [35]. The study found that while initial resistance to workstation crowding existed, nurses eventually adapted to the new arrangement and appreciated its benefits, such as reducing cross-contamination and improving job satisfaction [35]. This issue also shows that with proper training and support, nurses can accept and benefit from measures designed to prevent the spread of COVID-19 [35]. Reducing workstation crowding is crucial in preventing the spread of infectious diseases and pandemics. Social distancing, mask-wearing, and proper ventilation can improve the work environment and minimize disease transmission.

Limiting visits to healthcare facilities to control the spread of infectious diseases such as COVID-19 is crucial. One of the essential strategies that most nurses adopt is to restrict access and meetings. According to a study by Martel et al., such measures can significantly reduce the risk of virus transmission [36]. The study further revealed that separating visitors from patients could substantially reduce the spread of respiratory viruses, including COVID-19 [36]. This approach requires careful planning to ensure patients and visitors communicate effectively without compromising safety. According to a study by Sarah et al., nurses generally support limiting visits to prevent the spread of COVID-19 despite concerns about practicality and feasibility [37]. To successfully implement these strategies, open communication and consultation with nurses is crucial to address their concerns. Limiting appointments is a practical approach to preventing the spread of infectious diseases [37]. Reducing the number of appointments and close contact between people can decrease the possibility of disease transmission. This action can help control the spread of diseases and prevent further transmission. Video meetings and phone calls can be used to discuss and exchange information virtually while reducing the risk of disease transmission.

Shortening work sessions or shifts can help prevent the spread of the pandemic and reduce infections. A study showed that shorter work days and longer breaks can increase job satisfaction and reduce work-related diseases, effectively managing the epidemic burden on healthcare workers [38]. Reducing work sessions or shifts can control COVID-19 spread. Fewer meetings and shifts lower contact, enabling social distancing. This action reduces cases, helps prevent the spread, and improves society's health.

Most participants in a recent study followed health guidelines to cope with the risk of contracting the virus. Adequate training helped nurses to use personal protective equipment (PPE) and follow infection control practices consistently. Support from colleagues and leadership encouraged safety protocols and reporting of concerns. However, some nurses fear contracting the virus and may consider taking leave or changing jobs [39]. This study emphasizes the importance of addressing healthcare workers' fear of contracting the virus during outbreaks. Acknowledging their concerns is crucial in responding appropriately to the COVID-19 pandemic. Healthcare institutions must provide adequate support and resources, including training, PPE, and emotional support, to help nurses cope with the risks associated with their work environment. Workers exhibiting cold/flu symptoms should stay home to prevent disease transmission. Nurses, in particular, prefer to avoid communication with symptomatic colleagues at work. Avoiding contact in the workplace can reduce transmission, and those with symptoms should wear masks and maintain social distancing. Healthcare organizations should reinforce sick leave policies and prioritize worker/patient well-being through training, communication, and supportive policies.

Employees with cold or flu symptoms should stay home to prevent the spread of illnesses. Nurses typically avoid contact with colleagues who show symptoms at work [40]. To limit the transmission of diseases in the workplace, staying away from coworkers exhibiting symptoms and following prevention protocols, such as wearing a mask and practicing social distancing, is recommended. It is crucial to emphasize the importance of adhering to sick leave policies as a public health concern through regular training, clear communication, and supportive policies that prioritize the well-being of healthcare workers and patients.

Some participants may question the necessity of taking COVID-19 precautions. However, many nurses agree that personal precautions are crucial [41]. Providing training, accurate information, and creating open communication channels are essential to promoting coordination among healthcare workers [42]. It is vital to recognize the psychological impact of the pandemic on healthcare workers and provide them with supportive work environments. Additionally, it is crucial to communicate safety measures and ensure that everyone follows them.

A study found that healthcare professionals feel their hospitals must prepare for pandemics and are not involved in decision-making. Involving employees in decision-making and preparation processes is crucial for maintaining morale and quality of care during such events [43]. It is essential to create supportive and inclusive work environments that recognize the critical role of healthcare professionals in managing the healthcare system during a pandemic. The hospital organization should ensure the preparation and necessary equipment for the pandemic and support employee participation in the preparation process.

The COVID-19 pandemic has highlighted the need for nurses to be well-prepared to handle future epidemics and similar cases. Identifying the problems nurses face can lead to better planning, preparation, and management [44]. The following recommendations are suggested to better equip nurses to respond to viral outbreaks and pandemics. Firstly, it is essential to increase nurses' awareness of hand hygiene and the proper use of personal protective equipment through appropriate educational programs. Secondly, accurate information about influenza and COVID-19 vaccinations must be promoted, and nurses should be encouraged to get vaccinated. Thirdly, health and wellness programs for nurses and healthcare professionals should be implemented to enhance their skills and practices. Finally, facilitating remote work for nurses can help maintain their health and reduce the risk of infection transmission.

## Limitation

This study aimed to evaluate the readiness of Iranian nurses to face potential pandemic outbreaks. However, the study has certain limitations. Firstly, it was conducted only in Ardabil, Iran; thus, its findings may only apply in some countries with different cultural and social conditions. Secondly, the study relied solely on self-reported questionnaires, possibly leading to biased results. Therefore, it is crucial to interpret the results with caution. To obtain more accurate and reliable results, researchers should use diverse data collection methods, including interviews alongside self-reported questionnaires. Additionally, conducting multi-center studies across various regions can improve the study's applicability beyond a single location. By adopting these strategies, future studies can provide more comprehensive insights into the preparedness of healthcare professionals in facing potential pandemic outbreaks.

## Conclusion

Healthcare providers, especially nurses, play a crucial role in mitigating the impact of disasters on victims and the healthcare system [45]. Our study has found that 90.1% of the surveyed nurses consider hand washing the most effective measure to prevent spreading infections. Screening for COVID-19 in the workplace and implementing health and wellness programs for healthcare workers were also identified as effective methods to combat the pandemic. Telecommuting was found to help reduce the spread of the pandemic among nurses. Nurses have efficiently utilized the COVID-19 Safe program to respond to the pandemic and assist infected individuals. Social media platforms and visual aids such as posters were valuable in disseminating COVID-19 information among healthcare professionals. Continuous education and promotion of proper hand hygiene practices among nurses are essential to strengthening infection prevention measures. Additionally, implementing health and wellness programs can improve diagnostic capabilities, enhance performance, and mitigate fatigue and stress-related risks. Remote work facilities can ensure nurses' health and reduce infection transmission risks. Utilization of the COVID-19 Safe program can result in an effective pandemic response and maintain healthcare professionals' well-being. These strategies aim to empower nurses in infection control, promote workplace safety, and optimize patient care outcomes during the pandemic.

## Data Availability

The datasets used and analyzed during the current study are available from the corresponding author upon reasonable request.

## References

[1] Huh S (2020). How to train health personnel to protect themselves from SARS-CoV-2 (novel coronavirus) infection when caring for a patient or suspected case. J Educ Eval Health Prof.

[2] Najafi M, Nazari M, Pouragha B, Mohammadi AJ, Baghian N, Ashrafi E, Rajaee R (2021). Studying COVID-19 disease management: A review study.

[3] Lai C-C, Shih T-P, Ko W-C, Tang H-J, Hsueh P-R (2020). Severe acute respiratory syndrome coronavirus 2 (SARS-CoV-2) and coronavirus disease-2019 (COVID-19): The epidemic and the challenges. Int J Antimicrob Agents.

[4] Vatandoost V, Tabatabaee SS, Okhovati M, Barooni M (2023). Explaining the challenges of resources management and its underlying factors in COVID-19 era in Iran: a qualitative study. BMC Public Health.

[5] Team IC-HSUF, Murray CJ: Forecasting COVID-19 impact on hospital bed-days, ICU-days, ventilator-days and deaths by US state in the next 4 months. MedRxiv 2020:2020.2003. 2027.20043752.

[6] Søreide K, Hallet J, Matthews JB, Schnitzbauer AA, Line PD, Lai PB, Otero J, Callegaro D, Warner SG, Baxter NN (2020). Immediate and long-term impact of the COVID-19 pandemic on delivery of surgical services. Br J Surg.

[7] Li Q, Guan X, Wu P, Wang X, Zhou L, Tong Y, Ren R, Leung KS, Lau EH, Wong JY (2020). Early transmission dynamics in Wuhan, China, of novel coronavirus–infected pneumonia. N Engl J Med.

[8] Tabatabaee SS, Vatandoost V, Saghi FK, Daghighbin E (2022). Explaining the challenges of hospitals admitting COVID-19 patients from the perspective of managers (a qualitative study). J Educ Health Promot.

[9] Adelaja I, Sayma M, Walton H, McLachlan G, de Boisanger J, Bartlett-Pestell S, Roche E, Gandhi V, Wilson GJ, Brookes Z (2020). A comprehensive hospital agile preparedness (CHAPs) tool for pandemic preparedness, based on the COVID-19 experience. Future Healthc J.

[10] Scanlon DA, Murphy DM, Smolowitz DJ, Lewis DV (2023). Alignment of the International Council of Nurses Advanced Practice Nursing Guideline Characteristics With Conceptual Frameworks: A Scoping Review. Res Theory Nurs Pract.

[11] Shrestha GS, Paneru HR, Acharya SP, Shrestha SK, Sigdel MR, Tiwari S, Yadav BK, Rijal B, Karki L, Neupane Y (2020). Preparedness for coronavirus disease in hospitals of Nepal: a nationwide survey. JNMA J Nepal Med Assoc.

[12] Akalu Y, Ayelign B, Molla MD (2020). Knowledge, attitude and practice towards COVID-19 among chronic disease patients at Addis Zemen Hospital. Northwest Ethiopia Infect Drug Resist.

[13] Wang D, Hu B, Hu C, Zhu F, Liu X, Zhang J, Wang B, Xiang H, Cheng Z, Xiong Y (2020). Clinical characteristics of 138 hospitalized patients with 2019 novel coronavirus–infected pneumonia in Wuhan. China JAMA.

[14] Kline RB. Beyond significance testing: Statistics reform in the behavioral sciences. American Psychological Association. 2013.

[15] McGill N, Weller-Newton J, Lees C (2022). A new survey tool for evaluating pandemic preparedness in health services. BMC Health Serv Res.

[16] Huang F, Armando M, Dufau S, Florea O, Brouqui P, Boudjema S (2021). COVID-19 outbreak and healthcare worker behavioural change toward hand hygiene practices. J Hosp Infect.

[17] Elhadi M, Msherghi A, Alkeelani M, Zorgani A, Zaid A, Alsuyihili A, Buzreg A, Ahmed H, Elhadi A, Khaled A (2020). Assessment of healthcare workers’ levels of preparedness and awareness regarding COVID-19 infection in low-resource settings. Am J Trop Med Hyg.

[18] Kwok KO, Li K-K, Wei WI, Tang A, Wong SYS, Lee SS (2021). Influenza vaccine uptake, COVID-19 vaccination intention and vaccine hesitancy among nurses: A survey. Int J Nurs Stud.

[19] Wang K, Wong ELY, Ho KF, Cheung AWL, Chan EYY, Yeoh EK, Wong SYS (2020). Intention of nurses to accept coronavirus disease 2019 vaccination and change of intention to accept seasonal influenza vaccination during the coronavirus disease 2019 pandemic: A cross-sectional survey. Vaccine.

[20] Grochowska M, Ratajczak A, Zdunek G, Adamiec A, Waszkiewicz P, Feleszko W (2021). A comparison of the level of acceptance and hesitancy towards the influenza vaccine and the forthcoming COVID-19 vaccine in the medical community. Vaccines.

[21] Al Baalharith IM, Pappiya EM (2021). Nurses' preparedness and response to COVID-19. Int J Afr Nurs Sci.

[22] Rivett L, Sridhar S, Sparkes D, Routledge M, Jones NK, Forrest S, Young J, Pereira-Dias J, Hamilton WL, Ferris M (2020). Screening of healthcare workers for SARS-CoV-2 highlights the role of asymptomatic carriage in COVID-19 transmission. Elife.

[23] Puylaert CA, Scheijmans JC, Borgstein AB, Andeweg CS, Bartels-Rutten A, Beets GL, van Berge Henegouwen MI, Braak SJ, Couvreur R, Daams F (2020). Yield of screening for COVID-19 in asymptomatic patients before elective or emergency surgery using chest CT and RT-PCR (SCOUT): multicenter study. Ann Surg.

[24] Korman MB, Steinberg R, Gagliardi L, Stewart B, Acero CL, Davies J, Maunder R, Walker T, DasGupta T, DiProspero L (2022). Implementing the STEADY Wellness Program to Support Healthcare Workers throughout the COVID-19 Pandemic. Healthcare.

[25] Bailey BC, Cox S, Terris L, van Oppen D, Howsare J, Berry JH, Winstanley EL (2023). Rural health care worker wellness during COVID-19: Compassion fatigue, compassion satisfaction & utilization of wellness resources. PLoS ONE.

[26] Hughes L, Petrella A, Phillips N, Taylor RM (2022). Virtual care and the impact of COVID-19 on nursing: A single centre evaluation. J Advanced Nurs.

[27] Birimoglu Okuyan C, Begen MA (2022). Working from home during the COVID-19 pandemic, its effects on health, and recommendations: The pandemic and beyond. Perspect Psychiatr Care.

[28] Almagor J, Picascia S (2020). Exploring the effectiveness of a COVID-19 contact tracing app using an agent-based model. Sci Rep.

[29] Vogt F, Haire B, Selvey L, Katelaris AL, Kaldor J (2022). Effectiveness evaluation of digital contact tracing for COVID-19 in New South Wales. Australia Lancet Public Health.

[30] Neely S, Eldredge C, Sanders R (2021). Health information seeking behaviors on social media during the COVID-19 pandemic among American social networking site users: survey study. J med Internet Res.

[31] Chan AK, Nickson CP, Rudolph JW, Lee A, Joynt GM (2020). Social media for rapid knowledge dissemination: early experience from the COVID-19 pandemic. Anaesthesia.

[32] Erinoso O, Wright KO, Anya S, Kuyinu Y, Abdur-Razzaq H, Adewuya A (2021). Predictors of COVID-19 information sources and their perceived accuracy in Nigeria: online cross-sectional study. JMIR Public Health Surveill.

[33] Tripathi R, Alqahtani SS, Albarraq AA, Meraya AM, Tripathi P, Banji D, Alshahrani S, Ahsan W, Alnakhli FM (2020). Awareness and preparedness of COVID-19 outbreak among healthcare workers and other residents of South-West Saudi Arabia: a cross-sectional survey. Front Public Health.

[34] Dancer SJ (2021). Reducing the risk of COVID-19 transmission in hospitals: focus on additional infection control strategies. Surgery (Oxf).

[35] Christie A, Brooks JT, Hicks LA, Sauber-Schatz EK, Yoder JS, Honein MA (2021). COVID C, Team R: Guidance for implementing COVID-19 prevention strategies in the context of varying community transmission levels and vaccination coverage. MMWR Morb Mortal Wkly Rep.

[36] Martel J, Dembicki J, Bubric K. Applying Human Factors to Modify and Design Healthcare Workspaces and Processes in Response to COVID-19. Ergonomics in Design. 2023;10648046231155949.

[37] Jordan SR, Connors SC, Mastalerz KA (2022). Frontline healthcare workers’ perspectives on interprofessional teamwork during COVID-19. J Interprof Educ Pract.

[38] Talic S, Shah S, Wild H, Gasevic D, Maharaj A, Ademi Z, Li X, Xu W, Mesa-Eguiagaray I, Rostron J (2021). Effectiveness of public health measures in reducing the incidence of covid-19, SARS-CoV-2 transmission, and covid-19 mortality: systematic review and meta-analysis. BMJ.

[39] Sperling D (2021). Nurses’ challenges, concerns and unfair requirements during the COVID-19 outbreak. Nurs Ethics.

[40] Tartari E, Saris K, Kenters N, Marimuthu K, Widmer A, Collignon P, Cheng VC, Wong SC, Gottlieb T, Tambyah PA (2020). Not sick enough to worry?" Influenza-like" symptoms and work-related behavior among healthcare workers and other professionals: Results of a global survey. PLoS ONE.

[41] Cengiz Z, Isik K, Gurdap Z, Yayan EH (2021). Behaviours and experiences of nurses during the COVID-19 pandemic in Turkey: A mixed methods study. J Nurs Manag.

[42] Kokabisaghi F, Akhtar F, Taghipour A, Javan-Noughabi J, Moghri J, Tabatabaee SS (2023). Why healthcare providers are not vaccinated? A qualitative study during the COVID-19 pandemic in Iran. BMC Prim Care.

[43] Laugesen B, Albrechtsen MT, Grønkjær M, Kusk KH, Nielsen MG, Jørgensen L, Pedersen B, Lerbæk B, Haslund-Thomsen H, Thorup CB (2022). Nurses’ clinical decision-making in a changed COVID-19 work environment: A focus group study. Global Qual Nurs Res.

[44] Farahmandnia H, Pourvakhshoori N, Abdolahi M, Molavi-Taleghani Y, Ziapour A. A. Experiences and Challenges of Nursing Managers’ Preparedness for Timing Response to COVID-19 Pandemic: A Qualitative Study in Iran. J Afr Nurs Midwifery. 2022;24.

[45] Farokhzadian J, Mangolian Shahrbabaki P, Farahmandnia H, Taskiran Eskici G, Soltani GF (2024). Nurses’ challenges for disaster response: a qualitative study. BMC Emerge Med.

